# 

**Published:** 1888-06-09

**Authors:** 


### CONTENTS.

**A Homily for Hospital Sunday Reading** - - 157

**Preachers and Subjects for Hospital Sunday** - 158

**A Few Words about our Hospitals.** By MRS. GLADSTONE 159

**Patients' Contributions to Hospitals.** By Mr. W. BURLETT-COUTTS, M.P. - - - - - - 161

**A Census of Hospital Workers** - - - - 162

**In and Out Among the Hospitals.** By a SECRETARY OF TEN YEARS' STANDING.—Pennsylvania Hospital.—The Middlesex Hospital.—Italian Hospital - - - - 163

**The Doctor's Pulpit.**—A Fresh Start with the Summer - 160

**Notes and News** - - - - - - - 164

**Everybody's Page** - - - - - - - 166

**Annotations** - - - - - - - - 167

**An Ishmalite.** By LESLIE KEITH, Author of "The Chilcotes," "East and West," etc. - - - - - - 163

**Dr. Bristowe on the Registration of Nurses** - 170

**The Editor's Letter-box** - - - - - 171

**Amusements with Profit for all Ages** - - - 172

**EXTRA SUPPLEMENT.—THE NURSING MIRROR.**

En Passant.—Workhouse Infirmary Nursing Association.—The "Sunday at Home"—Amateur Work.—A Brave Hospital Nurse.—News from Worcester.—Women Orators - xxxiii
The National Pension Fund. By Dr. J. S. BRISTOWE, F.R.S., Senior Physician to St. Thomas' Hospital - - - xxxiv
Everybody's Opinion - - - - - - - xxxviii
Anecdotes of Personal Adventure - - - - - xxxix
Original Articles.—Nursing the Insane - - - - xxxix
Examination Questions.—Notes and Queries.—Appointments.—Vacancies.—Exchange and Mart - - - - - xl

